# 

**Published:** 1850-07-01

**Authors:** 


### APPOINTMENT.

WE understand that *Dr. Hitchman*, who has for the last five years acted as resident medical officer at Hanwell Lunatic Asylum, on the female side of the establishment, has recently been elected to the office of *Superintendent Physician* to the Derby County Asylum, now being erected. We congratulate the committee of that asylum on having selected so able a man to preside over their new institution. Dr. Hitchman has paid much attention to the study of insanity, and his opportunities of acquiring a practical knowledge of the malady have been great. He has been associated with the visiting physician of Hanwell in giving clinical lectures at the institution. These lectures contain a vast fund of valuable information, and do great credit to Dr. Hitchman. It will be difficult to find an efficient substitute for him at the Hanwell Asylum.

---

### To Correspondents.

WE must crave the kind indulgence of our numerous correspondents. We are compelled to omit our usual list of books; and we reluctantly postpone our notice of the Reports of British and Continental Asylums, which have been forwarded to us. In our next Number, we hope to make ample amends for all unavoidable omissions.

